# Medical Society of Tennessee

**Published:** 1846-08

**Authors:** 


### THE WESTERN JOURNAL.

NEW SERIES.—VOL. VI.—NO. II.

LOUISVILLE, AUGUST 1, 1846.

### MEDICAL SOCIETY OF TENNESSEE.

It was with unfeigned pleasure that we received, a few days ago, a pamphlet containing the Proceedings of the Medical Society of Tennessee at the seventeenth annual meeting, held in Nashville in May last. This Society is growing almost venerable; it has lived a good way beyond a quarter of a century, which is a long period for institutions in our country founded upon the voluntary principle. But, we trust, it has many years of usefulness still before it, and will go on to the end of its century without evincing any signs of decay.

The officers elected at the late meeting of the Society were—A. H. Buchanan, President; Daniel McPhail, Vice President; R. Martin, Treasurer; C. K. Winston, Corresponding Secretary; and J. W. Stout, Recording Secretary.

Among the medical reports made to the Society were, a case by Dr. Felix Robertson, of ulceration of the bladder, by which a communication between that viscus and the rectum was established; a case, by Dr. R. Martin, of an acephalous monster; a case, by Dr.

A. W. Nelson, of removal of the condyle and a portion of the ramus of the inferior maxillary bone; a case, by Dr. Irwin, of injury of the spine; a case, by Dr. Manlove, presenting all the symptoms of hydrophobia which he attributed to lead poison; a case, by Dr. Overton, said to be "well authenticated," of hydrophobia cured by a strong decoction of phytolacca decandra, and a case of the same disease, by Dr. Robertson, cured by cantharides given to the extent of exciting strangury. These reports will be looked for with interest by the profession, and, no doubt, will soon be made public.

At the meeting of the Society in 1845 a prize of fifty dollars was offered for the best essay on *Scrofula*. This, it appears from the printed proceedings, drew forth four essays, the most meritorious of which was pronounced to be one written by W. L. Sutton, M. D., of Georgetown, Ky. We shall have the pleasure, we presume, of laying it before our readers, as by an order of the Society it is to be forwarded to us for publication. Dr. Sutton, it will be remembered, was successful, a year ago, in winning the prize offered by a lady for the best essay on the "Health of Clergymen." The practice of tendering premiums for papers on medical subjects of special interest is a good one, and it is pleasing to see that it is beginning generally to prevail among the medical societies. The Louisiana Medico-Chirurgical Society offer a gold medal, of the value of one hundred dollars, for the best essay on *Strictures of the Urethra*, to be awarded in 1847. Last year a prize was offered by the Medical Society of Alabama for the best essay on *Congestive Fever*, which was taken by Dr. Mabry, of Selma. The same Society is now offering a prize for the best *Medical History of Alabama*. The medical talent of the country will be brought out by these calls.

